# Heroism

**Published:** 1887-06-18

**Authors:** 


### HEROISM.

Risking one's life for country, friend, or foe,
Or, as in older times, for Holy faith;
Is heroism of the noblest kind.
But there are other types of humbler sort,
These, too, may some day earn the martyr's crown.
Forgetting self in the small things of life,
Bearing with cheerfulness the sharpest pain,
Suffering meekly taunts of cruel words,
Preferring others' good to selfish gain,
All this is work for heroes! heroines!—K. M.

